# The Use of Arsenic in Pulmonary Tuberculosis

**Published:** 1885-11

**Authors:** 


### The Use of Arsenic in Pulmonary Tuberculosis.

According to the "Centfalblatt fur klinische Medicin," Stinzing has tried the arsenic treatment at von Ziemssen's clinic in sixteen cases, two of which have proved fatal. In one of these cases the drug could be employed only for eleven days, and in the other only for three weeks. In but four cases was there any noticeable lowering of the temperature ; in eight it was entirely unaffected ; and in one case the drug even seemed to raise it. In three cases there had been no fever. In only one instance was there any diminution of the dyspnœa, of the cough, or of the expectoration. The frequency of the pulse was maintained in four cases, increased in six, and decreased in two. Nutrition was not increased in a single instance ; the weight of the body remained the same in two cases, and sank in nine, while two cases of apparent gain were accounted for by a highly dropsical condition. In no case was there a subsidence of the local processes in the lungs, but in eleven they advanced notably. The breathing capacity was repeatedly measured in twelve cases ; four of the patients improved a little in this respect, but this did not seem very certain, for, on account of the patients' awkwardness, the early measurements were thought to have been too low ; in two cases the capacity remained the same ; and in six it was evidently diminished. The number of bacilli found was unaffected in seven cases, and increased in three ; in only one was it diminished, and that several weeks after the use of the remedy had been discontinued.

The Government astronomer of Hong-kong has published an account of the phenomena which precede typhoons. The first signs are feathery clouds in the sky of the cirrus type, looking like fine tufts of white wool, and which travel from east to north. These appearances are accompanied by a slight rise of the barometer, clear weather, heat, and light winds. The barometer then begins to fall ; the heat becomes oppressive ; there is a swell on the sea, and the sky assumes a threatening appearance. As the storm approaches these effects become more marked, while the wind gradually increases in force. Near the centre of the storm the wind blows with such violence that no canvas can hold against it, and the rain pours down in torrents. Still nearer the centre, the sea is lashed into such fury that this is the most dangerous position for ships. Typhoons are most common during September and August, but they are met with all the year round.

